# Association of Upper Limb and Gait Performance With Basic and Instrumental Activities of Daily Living in Patients With Multiple Sclerosis

**DOI:** 10.7759/cureus.104670

**Published:** 2026-03-04

**Authors:** Selma Sabanagic-Hajric, Nevena Mahmutbegovic, Amar Hadzagic, Elhana Maric, Merima Unkic, Amina Zorlak-Cavcic

**Affiliations:** 1 Neurology, Clinical Center of the University of Sarajevo, Sarajevo, BIH; 2 Neurology, General Hospital “Prim. Dr. Abdulah Nakaš”, Sarajevo, BIH; 3 Forensic Medicine, Faculty of Medicine, University of Sarajevo, Sarajevo, BIH

**Keywords:** activities of daily living, functional independence, motor skills, multiple sclerosis, rehabilitation

## Abstract

Objective

This study aimed to examine the associations between objective functional performance tests, clinical disability, MRI lesion characteristics, and recent relapse activity with basic and instrumental activities of daily living (ADLs/IADLs) in people with multiple sclerosis (MS).

Materials and methods

In this cross-sectional study including 65 patients with MS, clinical disability was assessed using the Expanded Disability Status Scale (EDSS), while functional performance was evaluated with the Timed 25-Foot Walk test (T25FW) and the 9-Hole Peg Test (9-HPT). Functional independence was assessed using the Barthel Index for basic ADL and the Lawton IADL scale. MRI lesion characteristics and relapse activity during the preceding two years were recorded. Associations were analyzed using Spearman correlation and multivariable linear regression models.

Results

Higher EDSS scores and worse T25FW and 9-HPT performance were associated with lower Barthel and IADL scores. In adjusted models, T25FW remained independently associated with basic ADL, while non-dominant hand 9-HPT was the strongest independent predictor of IADL; EDSS showed a weaker independent association with IADL. MRI lesion variables and recent relapse activity were not independently associated with functional independence.

Conclusions

Simple performance-based measures of gait speed and upper limb dexterity are strongly associated with real-life functional independence in MS and may contribute to a more comprehensive functional assessment in routine clinical practice.

## Introduction

Multiple sclerosis (MS) is a chronic inflammatory and demyelinating disorder of the central nervous system with an immune-mediated pathogenesis, associated with a broad spectrum of neurological manifestations and variable levels of long-term disability. The disease most commonly affects young adults and remains one of the main non-traumatic causes of neurological disability in this age group [1]. Although the clinical presentation of MS is highly diverse, impairments of upper and lower limb motor function are among the most disabling features, substantially limiting patients’ ability to perform activities of daily living (ADLs) independently [2,3].

Because MS is a lifelong and often progressive condition, timely detection of motor dysfunction and estimation of its functional consequences are essential for individualized clinical decision-making and rehabilitation planning. The Expanded Disability Status Scale (EDSS) is widely used to quantify overall neurological disability; however, it does not adequately reflect performance in everyday functional tasks. For this reason, objective performance-based tests are increasingly applied to better describe the real-world functional impact of MS-related impairment [4]. In addition, the independent contribution of structural MRI findings and recent relapse activity to daily functional independence, beyond clinical performance measures, has not yet been fully clarified [5].

Standardized instruments, including the 9-Hole Peg Test (9-HPT) for upper limb dexterity, the Timed 25-Foot Walk test (T25FW) for gait performance, and the Lawton Instrumental Activities of Daily Living (IADL) scale for more complex daily tasks, offer complementary perspectives on functional status and everyday independence. Recent studies indicate that results of the 9-HPT and T25FW are not only reflective of current motor performance but may also be associated with functional independence in everyday life [6].

The primary objective of the study was to examine whether objective performance-based motor measures are independently associated with basic and instrumental functional independence in MS. Secondary exploratory objectives included evaluation of MRI lesion characteristics and recent relapse activity. To the best of our knowledge, this is the first study conducted in our region to jointly evaluate upper-limb dexterity and gait performance as independent correlates of basic and instrumental functional independence in people with MS.

## Materials and methods

Study design and setting

This cross-sectional study was conducted in 2025 at the Neurology Clinic, Clinical Center of the University of Sarajevo, Sarajevo, Bosnia and Herzegovina. Data collection was conducted between February and November 2025.

A total of 65 patients (n = 65) with a confirmed diagnosis of MS according to the 2017 revision of the McDonald diagnostic criteria were consecutively included. Eligibility criteria included age ≥18 years, a confirmed diagnosis of MS according to the 2017 McDonald criteria, clinical stability at the time of assessment, and ability to complete performance testing. Patients were excluded if they had an acute relapse or acute medical complication at assessment, severe cognitive or communication impairment preventing reliable testing, relevant comorbid neurological or musculoskeletal conditions affecting gait or upper-limb function, or incomplete key clinical/functional data. Sociodemographic and clinical variables (age, sex, disease duration, disease course, and current disease-modifying therapy) were recorded. Disability was assessed using the EDSS, and performance testing included the T25FW and the 9-HPT (dominant and non-dominant hand). The EDSS is a widely used scale ranging from 0 to 10, reflecting overall neurological disability in MS. The T25FW assesses short-distance walking speed as a measure of ambulatory function. The 9-HPT evaluates upper limb dexterity by measuring the time required to complete a standardized peg placement task.

Functional independence was assessed using the Barthel Index and the Lawton IADL scale. The Barthel Index measures independence in basic ADLs (range 0-100), while the Lawton IADL scale assesses more complex instrumental daily activities (range 0-8). The Lawton IADL scale was applied as a standardized clinical assessment tool to evaluate instrumental functional independence. The instrument was administered in routine clinical practice, and no copyrighted material, item descriptions, or scoring sheets were reproduced in the manuscript. The original publication of the scale was appropriately cited. Permission for reuse has been obtained via Oxford University Press Rights licensing service. All assessment instruments were used solely as outcome measures within clinical evaluation, and no proprietary content was reproduced.

Ethical approval

The study was conducted in accordance with the Declaration of Helsinki. The study protocol was approved by the Institutional Ethics Committee of the Clinical Center of the University of Sarajevo (approval no. 06-04-9-33476-5), and written informed consent was obtained from all participants prior to inclusion.

Statistical analysis

Descriptive statistics were used to summarize demographic, clinical, and functional variables. Distribution of continuous data was assessed using histogram inspection and the Shapiro-Wilk test; results are presented as mean ± standard deviation (SD) or median (interquartile range, IQR), as appropriate. Categorical variables are reported as frequencies and percentages.

Because EDSS is an ordinal scale and several outcome variables were non-normally distributed, associations between EDSS, performance tests (T25FW and 9-HPT), and functional independence measures (Barthel Index and Lawton IADL) were evaluated using Spearman’s rank correlation coefficient (ρ).

To examine independent relationships, separate multivariable linear regression models were fitted with the Barthel Index and Lawton IADL as dependent variables. Although EDSS is ordinal, it was modeled as a quasi-continuous covariate, consistent with common practice in MS research, primarily to adjust for overall disability burden while preserving clinical interpretability. All candidate predictors (EDSS, T25FW, non-dominant hand 9-HPT, juxtacortical lesion category, spinal cord lesions, and relapse activity during the previous two years) were prespecified based on clinical relevance and entered simultaneously into the models. Standardized β coefficients are reported. Disease-modifying therapy was recorded descriptively but was not included in regression analyses due to treatment heterogeneity and limited sample size for subgroup evaluation.

Regression assumptions were evaluated prior to model interpretation. Linearity and homoscedasticity were assessed by visual inspection of standardized residual plots, and normality of residuals was examined using histograms and Q-Q plots. Multicollinearity was assessed using variance inflation factors (VIFs). Moderate collinearity was observed among clinically related variables, which is expected given overlapping disease severity constructs; however, all predictors were retained due to their established clinical relevance. Model performance was described using R^2^ and adjusted R^2^. Given the sample size relative to the number of predictors, findings should be interpreted with appropriate caution regarding potential model overfitting. Statistical significance was defined as p < 0.05 (two-sided). All analyses were performed using IBM SPSS Statistics for Windows, Version 26 (Released 2018; IBM Corp., Armonk, New York, United States).

## Results

Sociodemographic and clinical characteristics are presented in Table 1. A total of 65 patients with MS were included in the study. The mean age was 36.2 ± 9.2 years, and the majority of participants were female (45; 69.2%). The median disease duration was 2.0 years, with an overall mean disease duration of 3.22 ± 3.37 years. The most frequent disease course was relapsing-remitting MS (RRMS; 59, 90.8%), while secondary progressive MS was observed in five patients (7.7%), and primary progressive MS in one patient (1.5%).

**Table 1:** Sociodemographic and clinical characteristics of patients with MS Data are presented as mean ± standard deviation (SD) or frequency (%). MS: multiple sclerosis; EDSS: Expanded Disability Status Scale; RRMS: relapsing-remitting multiple sclerosis; SPMS: secondary progressive multiple sclerosis; PPMS: primary progressive multiple sclerosis; MRI: magnetic resonance imaging * Platform therapy includes interferon beta, glatiramer acetate, dimethyl fumarate, and teriflunomide; ^†^ High-efficacy therapy includes fingolimod, ocrelizumab, and siponimod.

Characteristic	N (%)/Mean ± SD
Age, years	36.2 ± 9.2
Female sex	45 (69.2)
Disease duration, years	3.22 ± 3.37
EDSS score	2.08 ± 1.70
MS disease course	
RRMS	59 (90.8)
SPMS	5 (7.7)
PPMS	1 (1.5)
Dominant hand	
Right-handed	59 (90.8)
Left-handed	6 (9.2)
Relapse activity in the last two years	
≥1 relapse	46 (70.8)
No relapses	19 (29.2)
Disease-modifying therapy	
No therapy	19 (29.2)
Platform therapy*	30 (46.2)
High-efficacy therapy^†^	16 (24.6)
MRI findings	
Spinal cord lesions present	17 (26.2)
No spinal cord lesions	48 (73.8)
Juxtacortical/cortical lesion category	
No juxtacortical lesions	14 (21.5)
Juxtacortical lesions without cortical lesions	22 (33.8)
Cortical lesions (with juxtacortical lesions)	29 (44.6)

The mean EDSS score was 2.08 ± 1.70, indicating predominantly mild to moderate neurological disability. Relapse activity within the previous two years was recorded in 46 patients (70.8%).

Regarding MRI findings, spinal cord lesions were present in 17 patients (26.2%). Juxtacortical and/or cortical lesions were identified in 29 patients (44.6%). Cortical lesions were documented only in the presence of accompanying juxtacortical lesions, in accordance with routine clinical MRI reporting practice at our institution.

Functional performance measures are summarized in Table 2. The mean completion time on the 9-HPT was 26.6 ± 8.2 seconds for the dominant hand and 28.3 ± 9.1 seconds for the non-dominant hand. The mean time required to complete the T25FW was 8.23 ± 2.50 seconds.

**Table 2:** Functional performance measures Data are presented as mean ± standard deviation (SD). 9-HPT: 9-Hole Peg Test; T25FW: Timed 25-Foot Walk Test

Functional measure	Mean ± SD	Median (IQR)
9-HPT - dominant hand, seconds	26.6 ± 8.2	23.5 (20.1-30.2)
9-HPT- non-dominant hand, seconds	28.3 ± 9.1	24.0 (21.9-33.1)
T25FW, seconds	8.23 ± 2.50	7.10 (6.64-8.90)
Barthel Index (0-100)	89.2 ± 11.7	95 (85-95)
Lawton Instrumental Activities of Daily Living (0-8)	6.51 ± 1.20	7 (6-7)

Functional independence in basic ADLs was high, with a mean Barthel Index score of 89.2 ± 11.7. In contrast, performance in IADLs was lower, with a mean Lawton IADL score of 6.51 ± 1.20, indicating greater limitations in more complex everyday activities.

Spearman correlation analyses between clinical variables and functional independence are presented in Table 3. Greater neurological disability, reflected by higher EDSS scores, was strongly associated with poorer functional independence, both for the Barthel Index (ρ = -0.88, p < 0.001) and for the Lawton IADL scale (ρ = -0.86, p < 0.001).

**Table 3:** Correlations between functional performance tests and independence measures *** p < 0.001 Data are presented as Spearman’s rank correlation coefficient (ρ) with corresponding p-values. EDSS: Expanded Disability Status Scale; 9-HPT: 9-Hole Peg Test; T25FW: Timed 25-Foot Walk Test; IADL: instrumental activities of daily living

Variable	Barthel Index	Lawton IADL
EDSS	-0.88***	-0.86***
T25FW, seconds	-0.78***	-0.71***
9-HPT - non-dominant hand, seconds	-0.78***	-0.78***

Slower gait performance on the T25FW was also significantly related to lower functional outcomes, showing negative correlations with the Barthel Index (ρ = -0.78, p < 0.001) and Lawton IADL scores (ρ = -0.71, p < 0.001). In addition, longer completion times on the 9-HPT for the non-dominant hand were strongly associated with reduced independence on both the Barthel Index (ρ = -0.78, p < 0.001) and the Lawton IADL scale (ρ = -0.78, p < 0.001).

The results of the multivariable linear regression analyses are summarized in Table 4. In the regression model with the Barthel Index as the outcome variable, greater neurological disability measured by EDSS (β = -0.48, p < 0.001), slower gait speed on the T25FW (β = -0.31, p = 0.002), and longer completion time on the non-dominant hand 9-HPT (β = -0.29, p = 0.004) remained independently associated with poorer performance in basic ADLs. The final model accounted for a large proportion of the variance in Barthel Index scores (adjusted R^2^ = 0.71).

**Table 4:** Multivariable linear regression models for functional independence in patients with multiple sclerosis Standardized β coefficients are reported with p-values. Adj. R^2^: adjusted coefficient of determination; 9-HPT: 9-Hole Peg Test; T25FW: Timed 25-Foot Walk Test; EDSS: Expanded Disability Status Scale; IADL: instrumental activities of daily living

Predictor	Barthel Index β (p-value)	Lawton IADL β (p-value)
EDSS	-0.48 (<0.001)	-0.33 (0.001)
T25FW, seconds	-0.31 (0.002)	-0.21 (0.018)
9-HPT - non-dominant hand, seconds	-0.29 (0.004)	-0.42 (<0.001)
Juxtacortical lesion category (0-2)	-0.08 (0.32)	-0.10 (0.26)
Spinal cord lesions (yes/no)	-0.06 (0.41)	-0.07 (0.39)
Relapse activity (≥1 in the last two years)	-0.05 (0.48)	-0.06 (0.44)
Model R^2^/Adjusted R^2^	0.74/0.71	0.69/0.66

For IADLs, the multivariable model identified non-dominant hand performance on the 9-HPT as the strongest independent contributor (β = -0.42, p < 0.001), followed by EDSS (β = -0.33, p = 0.001) and T25FW performance (β = -0.21, p = 0.018). This model explained 66% of the variability in Lawton IADL scores (adjusted R^2^ = 0.66).

Neither MRI lesion characteristics nor recent relapse activity showed an independent association with basic or instrumental functional independence after adjustment for clinical disability and functional performance measures.

## Discussion

This study shows that both upper limb dexterity and walking performance are closely related to everyday functioning in people with MS. After adjustment for relevant clinical variables, performance-based motor tests remained independently associated with functional independence in both basic ADLs and IADLs. Specifically, gait performance assessed by the T25FW was more strongly related to basic daily activities, whereas manual dexterity measured by the 9-HPT demonstrated the strongest association with IADLs. These findings highlight the clinical value of brief and easily applicable bedside motor assessments for identifying patients who are at risk of functional dependence and for supporting individualized rehabilitation planning in routine MS care.

Our results are in line with previous studies demonstrating the robustness and clinical relevance of these functional measures. The 9-HPT is a well-established tool for the evaluation of upper limb function and forms part of the Multiple Sclerosis Functional Composite because of its sensitivity to subtle changes in manual performance [7]. Similarly, the T25FW has been shown to be a practical and reliable indicator of ambulatory capacity and is widely used in both clinical trials and everyday practice [8].

Evidence from large observational cohorts further supports the prognostic relevance of gait assessment. Kalinowski et al. [9], using data from more than 47,000 T25FW evaluations within the Multiple Sclerosis Outcome Assessments Consortium (MSOAC) database, demonstrated excellent test-retest reliability and showed that slower walking speed was associated with subsequent disability progression, frequently preceding changes in EDSS scores. In addition, Groppo et al. [10] reported that deterioration in T25FW performance often occurred before EDSS worsening in patients with both primary and secondary progressive MS. In the present cohort, the strong relationship between walking speed and the Barthel Index emphasizes the importance of mobility for basic daily activities, whereas the weaker association with IADL after multivariable adjustment suggests that more complex daily tasks depend on additional functional domains beyond gait alone.

The importance of upper limb function for daily independence has also been emphasized in previous research. Solaro et al. [11] demonstrated significant associations between manual dexterity, hand strength, and functional autonomy in people with MS. Our findings extend these observations by showing that non-dominant hand performance on the 9-HPT was the strongest independent correlate of IADLs. This supports the concept that fine motor control plays a particularly important role in complex everyday tasks such as household management, communication, and independent living. Moreover, the independent association between 9-HPT performance and the Barthel Index indicates that upper limb dysfunction contributes not only to higher-level activities but also to limitations in basic self-care.

In the present study, both the Barthel Index and the Lawton IADL scale were used to capture different aspects of functional independence. Previous work by Aguilar-Zafra et al. [12] demonstrated high agreement and validity of the Barthel Index in MS populations, while Salter et al. [13], in a large international cohort, showed that the IADL scale is able to discriminate across disability levels and predict declining independence. Our results confirm that these two scales provide complementary information: the Barthel Index primarily reflects global physical disability and mobility, whereas IADL appears to be more sensitive to impairments in upper limb function and subtle functional restrictions that may not be fully reflected by EDSS scores. Clinically, such discrepancies are frequently observed in patients with relatively low EDSS values who nonetheless report difficulties in daily tasks requiring manual dexterity and sustained coordination.

Functional independence in MS is influenced by multiple interacting factors. Oliveira-Kumakura et al. [14] reported greater dependence among patients with longer disease duration and pyramidal involvement, as well as an adverse impact of socioeconomic characteristics on self-care and treatment adherence. Similarly, Månsson and Lexell [15] observed that even individuals with advanced MS may retain independence in basic personal activities while experiencing marked limitations in instrumental daily tasks. These observations are consistent with our findings, in which EDSS remained independently associated with the Barthel Index but showed a weaker relationship with IADL once objective performance measures were included. Furthermore, Jansa et al. [16] demonstrated that functional difficulties may occur across the entire EDSS spectrum, indicating that conventional disability scales may underestimate real-life limitations.

Beyond physical functioning, motor performance tests may also reflect broader aspects of disease impact. Mirmosayyeb et al. [17] reported that T25FW performance was more strongly related to cognitive status than EDSS, while 9-HPT showed significant associations with cognitive measures, although it did not remain an independent predictor. The strength of the associations observed in the regression models suggests that performance-based motor measures capture clinically meaningful dimensions of disability that are directly relevant to daily functioning. In particular, the higher standardized β coefficient of the non-dominant 9-HPT in the IADL model indicates a substantial contribution of fine motor control to complex real-world tasks. These findings support the notion that simple motor tests may capture multidimensional features of disability, extending beyond purely physical impairment.

In our multivariable analyses, neither MRI lesion characteristics nor recent relapse activity showed independent associations with functional independence. This finding is consistent with the well-recognized clinicoradiological paradox in MS and suggests that the influence of inflammatory activity and structural abnormalities on everyday functioning is largely mediated through clinical disability and functional performance rather than exerting a direct effect on daily independence [5,18]. The absence of independent associations between MRI lesion categories and functional independence may reflect the limited granularity of routine clinical MRI reporting, which does not capture lesion load, atrophy measures, or microstructural damage. Moreover, functional performance tests may better reflect integrated neural network dysfunction rather than isolated lesion localization.

Additional factors not systematically assessed in the present study may also contribute to reduced functional autonomy. Fatigue, as reported by Jaramillo-Buitrago and Pérez-Parra [19], was strongly associated with both basic ADLs and IADLs. Moreover, psychosocial and socioeconomic determinants have been shown to affect daily functioning and participation [13,14]. The absence of standardized assessment of fatigue, cognition, and psychosocial variables, therefore, represents an important limitation of this study.

Several limitations should be acknowledged. The relatively small sample size and single-center design restrict the generalizability of the findings. The cross-sectional nature of the study does not allow causal inference and limits conclusions regarding long-term predictive value. In addition, the cross-sectional design does not allow evaluation of treatment-related changes, improvement, or deterioration over time. Furthermore, MRI data were derived from routine clinical reports rather than quantitative lesion analyses, which may have reduced sensitivity for detecting more subtle structure-function relationships. Finally, the lack of longitudinal follow-up precludes evaluation of functional trajectories over time. Given the chronic and progressive nature of MS, longitudinal follow-up would be valuable to determine the predictive value of performance-based measures over time.

Despite these limitations, the functional assessments applied in this study are routinely used in clinical practice and are easy to administer, supporting the practical relevance of the present findings for everyday care of patients with MS.

## Conclusions

Despite its limitations, this cross-sectional study demonstrates that impaired fine motor performance and gait speed are strongly associated with reduced independence in daily functioning in patients with MS. Objective performance-based measures were independently related to both basic ADLs and IADLs, with upper limb dexterity showing particular relevance for instrumental functioning. Incorporating simple, standardized assessments of gait and upper limb function into routine clinical evaluation may contribute to a more comprehensive characterization of real-world functional status beyond conventional disability scales.
